# How Turbulence Brings Benefit: The Influence of Dynamic Team Environment on Entrepreneurial Team Innovation

**DOI:** 10.3389/fpsyg.2020.00759

**Published:** 2020-05-26

**Authors:** Xiao Deng, Ying Wang, Xiuli Sun

**Affiliations:** ^1^Business School, China University of Political Science and Law, Beijing, China; ^2^Guanghua School of Management, Peking University, Beijing, China

**Keywords:** dynamic team environment, entrepreneurial team innovation, uncertainty reduction theory, agreement-seeking behavior, team knowledge integration

## Abstract

From the perspective of entrepreneurial team processes, this study examines the effect of a dynamic team environment on entrepreneurial team innovation. Through applying uncertainty reduction theory, it proposes the influence mechanism and boundary condition of the relationship between dynamic team environment and entrepreneurial team innovation. By analyzing a sample of 270 entrepreneurial teams in China, it is found that a dynamic team environment can evoke entrepreneurial team innovation via triggering team members’ agreement-seeking behavior and then promoting team knowledge integration. In addition, team centralization of decision making can weaken the relationship between agreement-seeking behavior and team knowledge integration. Our findings contribute to a better understanding of entrepreneurial teams’ reactions to dynamic environments and the multistep mechanism that transfers the impact of a dynamic team environment to entrepreneurial team innovation through team members’ reactions and team interactions.

## Introduction

*“When force majeure strikes, the key to overcome lies in the people.”* — *Ren Zhengfei, CEO of Huawei.*

In the dynamic environment after the 2003 SARS outbreak, two entrepreneurial teams, Alibaba and JD.com, experienced huge challenges. To survive, they engaged in a series of innovations. Alibaba officially launched Taobao. JD switched its sales channel from the Zhongguancun counter to online communities. Ultimately, the innovations not only helped these two entrepreneurial teams survive but made a success of both Alibaba and JD. Entrepreneurial teams always confront a highly dynamic environment (Brouwer, 2000; Li and Atuahene-Gima, 2001). To outcompete under such conditions, keeping a high level of team innovation has been considered to be extremely important for entrepreneurial teams (George, 2007). However, how a dynamic environment can evoke entrepreneurial innovation remains unknown. Thus, this paper aims to explore how entrepreneurial teams react to the dynamic environment and seek advantages to improve team innovation.

Previous studies have done some work on this question. They found that organizational environmental dynamism can evoke organizational innovation by evoking strategic movement toward innovation (Freel, 2005). For example, some researchers propose that an uncertain or complex environment may bring in innovation through top managers deciding to take more aggressive venturing strategies (Özsomer et al., 1997). However, instead of increasing innovation from top-down organizational strategy, most entrepreneurs have reported that their team innovation is a bottom-up process and is generated from their team members and team interactions. Their team members provide creative solutions and new ideas to promote team innovation, and the whole team selects a really good one and implements it (Goman, 1989). Thus, in place of the traditional strategic perspective, we take the perspective of team processes, including team members’ reaction and interaction, to explore whether a dynamic team environment will and how it can influence entrepreneurial team innovation.

To do so, we apply uncertainty reduction theory from social psychology, which asserts that uncertainty reduction is an important motive for people’s specific behaviors (Knobloch, 1975). When facing uncertainty, people would like to react in ways that reduce the uncomfortable feelings brought by uncertainty (Knobloch, 1975), among which creating more consensus with other people can be especially effective. Thus, under a highly dynamic team environment, to reduce the potential hazard of uncertainty brought about by environmental dynamics, we propose that team members may react with sufficient communications to create more consensus between people, which has been conceptualized as agreement-seeking behavior by Knight et al. (1999).

**FIGURE 1:**
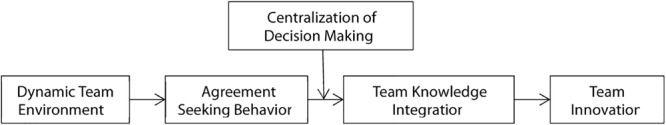
Research framework.

Previous studies emphasize that team members’ agreement-seeking behaviors can facilitate their communication, knowledge sharing, decision commitment, and discussion with each other in the team (Knight et al., 1999; Parayitam and Papenhausen, 2018; Mascareño et al., 2020). Thus, based on previous research, through entrepreneurial team members adopting more agreement-seeking behavior, we think team knowledge integration can be improved. Additionally, team knowledge integration has been shown to be a great help for increasing team innovation. Ultimately, team innovation can be affected. In general, to unveil the multistep mechanism between a dynamic entrepreneurial team environment and team innovation, we propose that a dynamic team environment will enhance team members’ agreement-seeking behavior (team members’ reaction), which will then improve team knowledge integration (team members’ interaction), and, ultimately, improve team innovation (team outcomes). Considering that entrepreneurial team structure can determine the effect of team members’ behavior on team members’ interaction, we also try to investigate the moderating effect of the centralization of the decision-making process. The hypothesized relationships are depicted in Figure 1.

Our theoretical constructions and empirical findings offer three significant contributions. Firstly, existing research takes a strategic perspective when exploring the effect of a dynamic environment on team innovation and ignores the fact that team members are the source of entrepreneurial team innovation (Hlavacek and Thompson, 1978). Through using a team processing perspective, this study reveals the black box inside an entrepreneurial team that can transfer the dynamic environment to team innovation and broadens the literature on how a dynamic environment can evoke team innovation. Secondly, by applying uncertainty reduction theory from social psychology, our work reveals the whole mechanism of how a dynamic team environment affects team members’ reactions, team interactions, and team outcomes step by step. Our research also shows how centralization of decision making (team structure) can be a moderator. Thirdly, most previous research only takes the team environment as a contingency factor instead of an antecedent factor for team processes and performance. The results of this research acknowledge the direct effect of the external environment on entrepreneurial teams, which enriches team environment research.

## Theoretical Background

A dynamic team environment refers to rapid, unpredictable, and turbulent change in the team environment (Eisenhardt and Mand Martin, 2000). Entrepreneurial teams with highly dynamic team environments will confront rapid and discontinuous change in market demand, competitors, technology, and/or regulation (Eisenhardt and Bourgeois, 1988; De Hoogh et al., 2004). In such an environment, entrepreneurial team members may perceive very high levels of uncertainty for two reasons (Tannert et al., 2007). On the one hand, because a lot of vital information is changing and unpredictable under a dynamic team environment (Lawrence and Lorsch, 1967), individuals may feel uncertain about team decisions and outcomes based on the lack of necessary information. Second, because past experiences may not apply and new things continuously show up under a dynamic team environment (Lawrence and Lorsch, 1967), entrepreneurial team members will feel uncertain about their capability to make good decisions.

Uncertainty may lead to undesired effects or significant loss. Thus, people always try their best to avoid uncertainty. The uncertainty reduction theory is one of the first theories to expose this phenomenon and asserts the notion that uncertainty reduction could be one of the leading motives behind human behaviors (Knobloch, 1975). When confronted with uncertainty, people tend to perform specific behaviors to decrease the uncertainty. This theory has already been applied in organizational study to explain the appearance of some specific behaviors such as feedback-seeking and humble leader behaviors (e.g., Morrison, 2002; Tuckey et al., 2002; Deng et al., 2019).

Based on uncertainty reduction theory, we think that a dynamic team environment can evoke agreement-seeking behaviors in members of an entrepreneurial team. Under such an environment, entrepreneurial team members will experience great uncertainty regarding decisions and outcomes owing to a lack of necessary information and be unconfident in decision-making (Lawrence and Lorsch, 1967). Previous research asserts that agreement-seeking behavior involves two efforts: the effort to discuss with others and the effort to reach a similar view of decisions (Knight et al., 1999). Both efforts in agreement-seeking behavior can be used to reduce uncertainty. On the one hand, through being widely involved in discussions, entrepreneurial team members can absorb more information from others, which can reduce uncertainty brought about by the lack of information. On the other hand, being involved in the process of reaching a similar view of decisions will cause entrepreneurial team members to make more evaluations of their thoughts, achieve consensus on final team decisions, and be more confident with the final decisions. Studies have also shown that when team members agree with each other, they tend to have more confidence in their decisions (Gero, 1985). Thus, by seeking agreement, entrepreneurial team members may obtain more information and believe in their final decision, and the uncertainty brought about by the lack of information and confidence can be reduced.

Therefore, to reduce the uncertainty brought about by a dynamic team environment, entrepreneurial team members under a dynamic team environment tend to apply more agreement-seeking behavior. We propose:

Hypothesis 1: The dynamism of the environment of an entrepreneurial team is positively related to entrepreneurial team members’ agreement-seeking behavior.

### The Mediating Roles of Team Agreement-Seeking Behavior and Team Knowledge Integration

The impetus for this section comes from the team process perspective, which states that team inputs can affect team members’ reactions and team interactions, leading to different team outcomes. In this section, following the team process perspective, we explore how a dynamic team environment (team inputs) evokes team agreement-seeking behavior (team members’ reactions), thereby increasing team knowledge integration (team interactions), which ultimately improves team innovation (team outcomes).

Team knowledge integration is a reliable pattern of team communication that generates joint contributions to the understanding of complex problems (Gardner et al., 2012; Sergeeva and Andreeva, 2016). Previous research stated that knowledge integration improves free and open sharing of ideas (Zand, 1972; Dirks, 1999). With a higher level of agreement-seeking behavior, team members share and exchange their own ideas more often to achieve committed decisions collectively in the decision-making process. During this process, by sharing, exchanging, and discussing team members ideas, team members’ knowledge can be integrated. Therefore, a higher level of agreement-seeking behavior can promote team knowledge integration.

For teams to be innovative, team members need to come up with creative ideas and critically process these ideas to abandon useless ones and keep promising ones (Amabile et al., 1996). Teams with higher knowledge integration have more members who directly participate in decision making and contribute with diverse information. Direct involvement helps team members understand complex problems and generate more creative thoughts. Additionally, cooperation on decision making brought by higher knowledge integration allows team members to become aware of the really good ideas and implement those rapidly (Campion et al., 1993). This process can enhance the team’s general ability to recognize the value of new information, apply it to commercial ends (Cohen and Levinthal, 1990), and implement good ideas. Thus, high team knowledge integration can improve team innovation by better generating and selecting new ideas.

As noted above, from a team process perspective, when team environments are very dynamic, team members tend to apply more agreement-seeking behavior in order to reduce uncertainty. The higher level of team agreement-seeking behavior will then increase team knowledge integration through more knowledge exchange and attempts to achieve consensus. With higher knowledge integration, team members can generate, recognize, and implement novel and useful ideas better, which ultimately improves the overall team innovation. By integrating these arguments, we come to our second hypothesis, which suggests that the effect of a dynamic team environment (team inputs) on team innovation (team outcomes) will be mediated by agreement-seeking behavior (team reactions) and team knowledge integration (team interactions):

Hypothesis 2: A dynamic team environment has a positive indirect effect on team innovation via team agreement-seeking behavior followed by team knowledge integration.

### The Moderating Role of Team Centralization of Decision Making

Centralization of decision making, which is one dimension of team structure, refers to the situation where the power of making truly influential decisions is confined to the higher levels of the hierarchy (Alexander and Fennell, 1986). In teams with high centralization of decision making, team leaders make the decisions, while, simultaneously, team members will be provided with fewer opportunities to be involved in decision making by the structural authority (Child, 1972). Conversely, in teams with low centralization of decision making, team members are offered more opportunities to be involved in team processes (Bunderson, 2003). Previous research has found that team structure can usually decide the extent of the effect of team member behaviors on their team interactions (Stewart and Stasser, 1995; Littlepage et al., 1997). When the team structure encourages the involvement of team members, the effect of team members on their team interactions will be increased. Thus, we propose that centralization of decision making, which is one dimension of team structure, can influence the relationship between team members’ agreement-seeking behavior and team knowledge integration.

In a team with high centralization of decision making, team members’ involvement and opportunities in group activities are severely constrained (Ibarra, 1992). Although they may have the tendency and willingness to share and contribute their own ideas to achieve collectively committed decisions, they are not really allowed or given a chance to do so (Joseph et al., 2016). As a result, team knowledge integration cannot be improved by team members’ agreement-seeking behavior. In contrast, in a decentralized decision-making team, where opportunities and rights to influence team decisions and actions are broadly distributed (Mookherjee, 2006), the team communication may be determined more by team members’ behaviors and characteristics through their participation (Chan et al., 1997). More entrepreneurial team members’ agreement-seeking behavior can be applied in the real decision-making process. Thus, entrepreneurial teams can achieve higher knowledge integration. Thus, we arrive at the hypothesis:

Hypothesis 3: Team centralization of decision making moderates the positive relationship between team agreement-seeking behavior and team knowledge integration such that this relationship will be weakened in the presence of high centralization of decision making.

## Materials and Methods

### Sample and Procedures

This study invited 290 team leaders and 889 team members working in 290 entrepreneurial teams from seven incubators in China to participate in this study. Among the seven incubators, three incubators are located in Beijing, two are in Anhui province, and two are in Hubei province. Almost all entrepreneurial teams are from Internet industries. We briefed the participants about the purpose of this study and explained the procedures for completing online surveys. To better protect the confidentiality of participants, this study assigned a random identification number to each participant to match leaders’ and team members’ responses after the survey.

To prevent common method bias, we collected the data in two waves with a 2-week interval each. In the first wave, the degrees of team environment dynamism and centralization of decision making were measured by team members. In the second wave, we invited 360 supervisors to rate their team innovation levels and received 290 responses, yielding a response rate of 80.6%. In addition, in the second wave, team members were asked to measure the extent of agreement-seeking behavior and team knowledge integration. In the end, 1,450 entrepreneurial team members were asked to provide their ratings, and 889 effective responses (61%) were collected that could be matched.

Since the study is based on the team level, we average the team member responses as the team level ratings of the variables mentioned above. A final sample of 290 teams was obtained. The size of each team ranged from 3 to 11 members, with an average of about 7.53 members. Prior to testing the hypotheses, we also conducted a confirmatory factor analysis (CFA) in AMOS 21.0 to demonstrate discriminant validity among our five latent constructs: dynamic team environment, agreement-seeking behavior, knowledge integration, team innovation, and centralization of decision making. The results showed a good fit to the data, with comparative fit index (CFI) = 0.974 and root-mean-square error of approximation (RMSEA) = 0.022.

### Measures

Seven-point Likert scales were used for all the variable measurements ranging from 1 = “strongly disagree” or “never” to 7 = “strongly agree” or “very frequently.” All the scales were first translated and then back-translated from English to Chinese to check for retention of the semantic content of the original questions.

#### Dynamic Team Environment

How dynamic the team environment is was assessed by team members with the adapted three-item scale (De Hoogh et al., 2004). A sample item is: “To which degree is your team environment dynamic?” Cronbach’s alpha for these questions was 0.879.

#### Agreement-Seeking Behavior

The measurement of agreement-seeking behavior was assessed by team members with a six-item scale (Knight et al., 1999). A representative item is: “Every team member believes that taking more time to reach consensus on a strategic decision is generally worth it.” Cronbach’s alpha for these questions was 0.928.

#### Team Knowledge Integration

Knowledge integration was evaluated by team members with a 10-item scale developed by Gardner et al. (2012). A sample item is: “Communications within our team were timely.” Cronbach’s alpha for these questions was 0.954.

Since all our variables were on the team level, all hypotheses were tested using one-level modeling. Single-level regression analysis was used to test Hypotheses 1. As Table 2 shows, the dynamism of the team environment was positively related to agreement-seeking behavior (β = 0.416, *p* < 0.001; Model 1). Thus, the result supports Hypothesis 1.

#### Team Innovation

The level of team innovation was rated by team leaders using the four-item scale developed by De Dreu and West (2001). A sample item is: “Team members often implement new ideas to improve the quality of products and services.” Cronbach’s alpha for these questions was 0.934.

#### Centralization of Decision Making

Centralization of decision making in the team was rated by team members using an adapted five-item scale (Dewar et al., 1980). A sample item is: “There can be little action taken here until a team leader approves a decision.” Cronbach’s alpha for these questions was 0.887.

#### Control Variables

Team size was treated as a control variable in our study based on previous research where team size is a key variable influencing team effectiveness and innovation (Brewer and Kramer, 1986). Team size was measured as the total number of team members within that team.

## Results

Descriptive statistics and correlations of different variables are presented in Table 1. The degree of dynamism in the team environment is significantly correlated with the extent of agreement-seeking behavior (β = 0.438, *p* < 0.01), team knowledge integration (β = 0.257, *p* < 0.01), and the level of team innovation (β = 0.356, *p* < 0.01).

**TABLE 1:** Descriptive statistics and correlations for study variables.

**Variable**	**Mean**	***SD***	**1**	**2**	**3**	**4**	**5**
1. Team size	7.53	1.75	–				
2. Dynamic team environment	5.15	1.18	0.156**	–			
3. Agreement-seeking behavior	5.08	1.20	0.149*	0.438**	–		
4. Knowledge integration	4.93	1.17	–0.094	0.257**	0.425**	–	
5. Team innovation	5.27	1.40	–0.099	0.356**	0.332**	0.434**	–
6. Centralization of decision making	4.07	1.71	−0.117*	−0.122*	–0.068	0.079	0.043

*N = 889*.*p < 0.05, ***p < 0.01, ***p < 0.001.

**TABLE 2:** Test of the study hypotheses.

	**Outcome: Agreement-**		**Outcome:**				**Outcome:**	
	**seeking behavior**		**Knowledge integration**				**Team innovation**	
	**Model 1**		**Model 2**		**Model 3**		**Model 4**	
	**γ**	**SE**	**γ**	**SE**	**γ**	**SE**	**γ**	**SE**
Team size	0.062	0.036	−0.114**	0.036	−0.106**	0.035	−0.099*	0.042
Dynamic work environment	0.416***	0.052	0.106	0.058	0.107	0.057	0.294***	0.068
Agreement-seeking behavior			0.394***	0.057	0.785***	0.1337	0.127	0.071
Centralization of decision making					0.572**	0.165		
Agreement-seeking behavior* centralization of decision making					−0.093**	0.030		
Knowledge integration							0.374***	0.068

N = 889. Unstandardized regression coefficients and unadjusted values are reported.*p < 0.05, **p < 0.01, ***p < 0.001.

To test Hypothesis 2, we use a Monte Carlo approach in Mplus. The Monte Carlo approach is recommended as a viable alternative to bootstrapping in complex multilevel models and models with multiple mediators (MacKinnon et al., 2004; Bauer et al., 2006; Preacher and Selig, 2012). To do so, first, we followed previous research and computed an estimate of this indirect effect as a product of three paths – dynamic team environment’s encouragement to agreement-seeking behavior (β = 0.416, *p* < 0.001; Model 1), agreement-seeking behavior’s influence on team knowledge integration (β = 0.394, *p* < 0.001; Model 2), and team knowledge integration’s effect on team innovation (β = 0.374, *p* < 0.001; Model 4). Second, with the help of Mplus, it was found, as shown in Table 3, that the total indirect effect from dynamic team environment on team innovation is 0.153 (*p* < 0.001). Also, the estimate of the specific indirect effect from dynamic team environment on team innovation through agreement-seeking behavior and knowledge integration is 0.063 (*p* < 0.001), which is positive and significant with a 99% confidence interval of [0.018; 0.108]. The result supports Hypothesis 2.

**TABLE 3:** Test of the indirect effect of dynamic team environment on team innovation.

	**Estimate**	**S.E.**	**Est/S.E.**	***P*-value**
Effects from dynamic team environment on team innovation				
Total	0.447	0.078	5.714	0.000
Total indirect	0.153	0.044	3.492	0.000
Specific indirect				
Team innovation				
Agreement-seeking behavior				
Dynamic team environment	0.057	0.032	1.764	0.078
Team innovation				
Team knowledge integration				
Dynamic team environment	0.033	0.029	1.111	0.266
Team innovation				
Team knowledge integration				
Agreement-seeking behavior				
Dynamic team environment	0.063	0.018	33.600	0.000

Besides, the specific indirect effect from dynamic team environment on team innovation only through agreement-seeking behavior is not significant (*p* = 0.078), and the specific indirect effect from dynamic team environment on team innovation only through knowledge integration is also not significant (*p* = 0.266). Thus, the result can imply that the indirect effect from dynamic team environment on team innovation can only be transferred by agreement-seeking behavior and team knowledge integration in sequence. It should also be noted that the direct effect of dynamic team environment on team innovation remains positive and significant when both mediators are in the model (β = 0.294, *p* < 0.001), which suggests that agreement-seeking behavior and team knowledge integration partially mediate the effect of dynamic team environment on team innovation.

**FIGURE 2:**
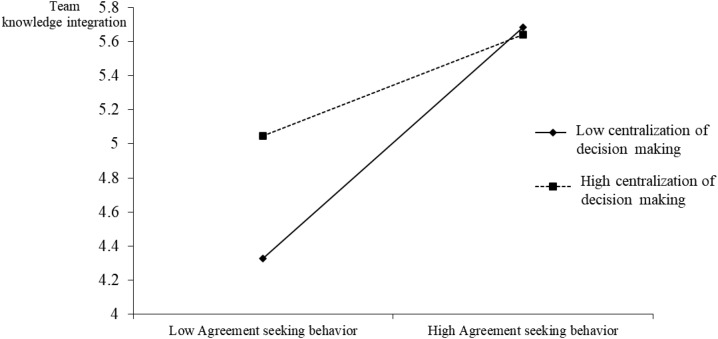
The interaction effect. Agreement-seeking behavior and centralization of decision making (High: Mean + 1sd; Low: Mean − 1sd).

Furthermore, the assumption of the interactive effect of agreement-seeking behavior and centralization of decision making on team knowledge integration in Hypothesis 3 is also significant (β = −0.093, *p* < 0.01). To test this interaction, we constructed an interaction plot and conducted a simple-slopes test (Aiken and West, 1991). As shown in Figure 2, the first simple slope computed for centralization of decision making at one standard deviation below the mean is positive and marginally significant (1.631, *p* = 0.091). The second simple slope computed for centralization of decision making at one standard deviation above the mean is negative but insignificant (−0.021, *p* = 0.990), suggesting that the moderating effect of centralization of decision making becomes more noticeable at its lower level.

## Discussion

In the two-time field study of entrepreneurial teams, we tested the hypotheses that environmental dynamism is positively related to team innovation through an increment in agreement-seeking behavior and team knowledge integration and that the positive relationship between agreement-seeking behavior and knowledge integration is weakened by the centralization of decision making.

### Theoretical Contributions

This research has several theoretical contributions. Firstly, we come up with a new perspective for exploring the relationship between environmental dynamism and team innovation. To explore the reason why a dynamic environment can evoke organizational innovation, most of the existing research starts from a strategic perspective (e.g., Courtney et al., 1997; Liang and Picken, 2010). In fact, team processes are a vital source for team innovation, but little attention has been paid from a team process perspective (Hargadon and Sutton, 2000; George, 2007; Amabile and Khaire, 2008; Cohn et al., 2009). Our work realizes the bottom-up path of how team innovation can be achieved. Additionally, we adopt the team process perspective to reveal the black box inside teams that relates dynamic team environments with entrepreneurial team innovation. We offer a new perspective for future studies.

Secondly, we creatively introduce uncertainty reduction theory to reveal the entire mechanism that imposes the effect of dynamic environments on team innovation. Specifically, by using uncertainty reduction theory, we find that a dynamic team environment can evoke team members’ agreement-seeking behavior to reduce their uncertainty about decisions and outcomes. Then, this behavior can improve team knowledge integration and team innovation. Our work introduces a new theory to the field of entrepreneurship and broadens the theory itself.

Thirdly, our results emphasize the direct influence of the external environment on entrepreneurial team processes and outcomes. Although previous works have acknowledged the importance of the team environment for team processes and outcomes, most of them only treated the external environment as a contextual or contingent factor (e.g., Hildreth, 1974; Tracey and Phillips, 2011). Few studies notice and prove the direct effect of the team environment. Because adaption to the external environment is vital for the survival and success of entrepreneurial teams, it is a good opportunity to test the direct effect of the external environment on team processes and outcomes. Additionally, our results prove the effect of a dynamic team environment on team members’ agreement-seeking behavior, team knowledge integration, and team innovation. Thus, our research can broaden environmental research.

### Limitations and Future Work

First, common method bias could still exist. In this study, agreement-seeking behavior and team knowledge integration were measured at the same time, and both were assessed by team members, which may cause a common method bias on the relationship between these two constructs. However, considering the fast change among entrepreneurial teams, we did not design the study with more time intervals of data collection. To eliminate probable alternative explanations, future studies are suggested to collect data from more different raters and at different times.

Second, our research provides only one kind of mechanism that explains the relationship between an uncertain environment and team innovation. Applying more processing variables to demonstrate the underlying mechanism is recommended. Furthermore, it would also be interesting to explore what potential negative effects environmental uncertainty may have on team innovation.

### Practical Implications

Entrepreneurs always try to stimulate team innovation under dynamic conditions to gain and maintain advantages over their competitors in the same industry (Ogilvie, 1998). Based on our empirical results, some important practical implications for entrepreneurial teams can be drawn. Firstly, the results suggest that agreement-seeking behavior and team knowledge integration are the antecedents of team innovation, which indicates that boosting these two kinds of behavior is more likely to improve team innovation. Practically, to improve team innovation, entrepreneurial teams can encourage and support team members’ agreement-seeking behavior and team knowledge integration. For example, every time before a meeting, entrepreneurs can set achieving consensus as the aim of the discussion. Also, entrepreneurs can act as a role model by seeking agreement in each discussion. Moreover, entrepreneurs can award those who try to seek agreement and integrate knowledge.

Secondly, our results support the hypothesis that low centralization of decision making can strengthen the positive relationship between agreement-seeking behavior and team knowledge integration. We suggest that entrepreneurs build up a more decentralized team structure in the team. Thus, team members would then have more chances to share, exchange, and contribute their ideas to the entrepreneurial team.

## Conclusion

Drawing on uncertainty reduction theory, we developed and empirically examined a model demonstrating the indirect effect of environmental uncertainty on team innovation from the team processing perspective. Our empirical results showed that perceived dynamic team environment is positively related to agreement-seeking behavior, which boosts team knowledge integration and later improves team innovation. Additionally, we showed that the positive relationship between agreement-seeking behavior and team knowledge integration is weakened by centralization of decision making. With the results, our study reveals the mechanism underlying the positive relationship between dynamic team environment and team innovation and provides empirical and practical insights into how entrepreneurial teams and entrepreneurs can improve team members’ innovation.

## Data Availability Statement

The original contributions presented in the study are included in the article/Supplementary File, further inquiries can be directed to the corresponding author/s.

## Ethics Statement

The studies involving human participants were reviewed and approved by Peking University. The patients/participants provided their written informed consent to participate in this study.

## Author Contributions

XD contributed to the project administration, conceptualization, data collecting, data analysis, and original draft preparation. YW contributed to the data collecting, data analysis, and, original draft preparation. XS contributed to the funding acquisition, original draft preparation, and reviewing and editing.

## Conflict of Interest

The authors declare that the research was conducted in the absence of any commercial or financial relationships that could be construed as a potential conflict of interest.

## Supplementary Material

FILE S1 Raw data.
